# Lysyl oxidase-like 2 promotes the survival, migration, and ferroptosis of endometrial cancer cells by activating the phosphoinositide 3-kinase/protein kinase B pathway

**DOI:** 10.22038/ijbms.2024.79933.17317

**Published:** 2025

**Authors:** Jiashi Gu, Huanmei Sun, Juan Shao, Hu Zhang, Zhanpeng Zhu, Dongqin Ma, Yingchun Duan

**Affiliations:** 1 Department of Obstetrics and Gynecology, Shanghai Pudong Hospital of Fudan University, Pudong, Shanghai-201399, China

**Keywords:** Apoptosis, Cell proliferation, Endometrial neoplasms, LOXL2 protein, Phosphorylation

## Abstract

**Objective(s):**

LOXL2, known as Lysyl oxidase-like 2, is classified as a lysyl oxidase (LOX) family member. However, its role and mechanism in endometrial cancer (EC) are unknown. Therefore, we aimed to investigate the potential role and mechanism of LOXL2 in EC.

**Materials and Methods:**

The levels of LOXL2 expression in EC tissues and normal adjacent tissues were evaluated by immunohistochemically (IHC) labeling. Following the dye application, 3-(4,5-dimethylthiazol-2-yl)-2,5-diphenyltetrazolium bromide (MTT) and Transwell methodologies were executed to evaluate the effects of LOXL2 inhibition and up-regulation on the growth, programmed cell death, migration, and susceptibility to iron-dependent cell death of EC. Moreover, protein analysis through Western blotting and gene expression analysis using Real-time quantitative PCR (RT-qPCR) was employed to measure the levels of pertinent biomarkers.

**Results:**

LOXL2 is highly expressed in both EC tissues and serum in vivo. Silencing LOXL2 reduced EC cell proliferation and migration while increasing apoptosis *in vitro*. LOXL2 silencing increased the ferroptosis-related proteins Solute Carrier Family 7 Member 11 (SLC7A11) and Ferritin Heavy Chain 1 (FTH1) while decreasing Glutathione Peroxidase 4 (GPX4) (both, *P<*0.001). Additionally, LOXL2 silencing reduced the p-PI3K and p-Akt protein expression, while LOXL2 overexpression (OE-LOXL2) elevated the p-PI3K and p-Akt protein expression (both, *P<*0.001). Additionally, LOXL2 silencing increases SLC7A11 and FTH1 while decreasing GPX4 (both *P<*0.001). LOXL2 overexpression has the opposite effect. However, the LY294002 inhibitor restores SLC7A11 and FTH1 expression while decreasing GPX4 (*P<*0.001).

**Conclusion:**

Our research demonstrated that LOXL2 might protect EC via phosphorylation by activating the PI3K/AKT pathway.

## Introduction

Endometrial carcinoma (EC) is the most frequent cancer in the field of gynecology, which poses a threat to the health and lives of women, consequently increasing the risk of morbidity and imposing a significant burden on the healthcare system and society (1). Most patients with EC can receive an early diagnosis and have a good prognosis (2). However, the prognosis remains poor for patients with advanced, poorly differentiated, or specific forms of EC, such as high-grade EC and EC with papillary serous or clear cell histology (3, 4). Even for those with early or well-differentiated EC, unexpected recurrence and a poor prognosis are common (5). Therefore, to improve the prognosis for patients with EC, it is crucial to investigate the pathophysiology of the disease and identify viable therapy targets.

A recently identified iron-dependent form of cell death is called ferroptosis. It is not the same as other types of cell death, such as necrosis, autophagy, and apoptosis. Iron metabolism, oxidative stress, and amino acid metabolism depend on ferroptosis. It is implicated in multiple physiological and pathological processes, such as immune response against viruses, ischemia-reperfusion injury, neuronal degeneration, and tumor suppression (6-9). Ferroptosis has been linked in studies to pancreatic cancer, liver cancer, stomach cancer, ovarian cancer, and breast cancer (10-15). For most tumors, ferroptosis activation has been considered a novel therapeutic strategy (16). However, it is not fully elucidated how ferroptosis and EC are related.

The lysyl oxidase (LOX) family includes lysyl oxidase-like 2 (LOXL2). Its principal function is to catalyze the extracellular matrix’s (ECM) elastin and collagen cross-linking (17). It also possesses certain intracellular functions linked to carcinogenic and fibrogenic effects (18). Numerous investigations have demonstrated that LOXL2 expression is markedly changed in several cancer types, including lung cancer, aggressive osteosarcoma, pancreatic ductal adenocarcinoma, prostate, colorectal, and breast cancer (19-24). It has a significant impact on the biological behavior of malignant tumors. Although the biological roles of LOXL2 in various cancer types have been established, the molecular mechanisms underlying LOXL2 in EC remain unclear.

A critical signaling pathway essential to cell functions is the PI3K/AKT pathway (25). This pathway is mediated by the phosphorylation of downstream substrates and is named after two key genes, PI3K and AKT (26). The primary function of this pathway is to promote metabolic activities, angiogenesis, growth, and cell survival in response to external stimuli (27, 28). However, the regulatory mechanism of the PI3K/AKT pathway in the survival, migration, and ferroptosis of EC cells remains unknown. Therefore, this research evaluated the potential impacts of LOXL2 on EC cells’ survival, migration, and ferroptosis and the underlying mechanisms.

## Materials and Methods

***Clinical specimens***

We collected five (5) cases of EC patients and obtained their cancer and normal adjacent tissues, which were surgically resected from July 2021 to December 2023 in our Hospital. We also collected serum samples from 15 EC patients and 15 healthy controls. The exclusion criteria are as follows: (1) Patients had primary EC and did not receive any radiotherapy or chemotherapy previously; (2) The postoperative tissues were diagnosed by pathologists as EC; patients were aware of and agreed to the whole process of the study; (3) Patients had complete clinical data and follow-up data. Exclusion criteria: (1) Patients with other malignant tumors; (2) Patients who had immune system diseases or infectious diseases; (3) Patients who had severe impairment of important organs, such as heart, liver, and kidney. Previously, none of the 20 patients with EC had received chemotherapy or radiation treatment. For hematoxylin and eosin (H&E) staining and immunohistochemistry, the cancer and normal adjacent tissues were fixed with 4% formaldehyde. They were then placed in liquid nitrogen storage for western blot and RT-qPCR. The ethical committee of Shanghai Pudong New Area Zhoupu Hospital approved using human tissue samples.

***Histology***

The histology procedure was performed using the method previously described (29). Skilled pathologists confirmed the histology of the EC and normal adjacent tissue samples after the samples were cut into 5 µm sections, fixed in 10% neutral buffered formalin, and stained with hematoxylin and eosin (H&E). 

***Immunohistochemistry analysis***

Target antigen distribution and location in cells or tissues can be determined using the immunohistochemistry (IHC) technique, which employs particular antibodies (30). In this study, EC tissues were sectioned into four μm pieces and embedded in paraffin. These sections were treated with 4% paraformaldehyde, stained with Masson’s trichrome, or treated with primary antibodies against LOXL2 (1:100; ab314140, Abcam) for 20 min at 37 ^°^C. Following the developer›s instructions, they were exposed to a biotinylated HRP-conjugated secondary antibody. Finally, the slides were examined using an optical microscope (IX51, Olympus, Japan).

***Enzyme-linked immunosorbent assay (ELISA)***

Serum samples were obtained from 15 EC patients and 15 healthy individuals. The amounts of LOXL2 (DY2639-05) in the samples were measured using an enzyme-linked immunosorbent assay (ELISA). The measurements were completed using ELISA kits from R&D Systems Inc. (Minneapolis, MN, USA) in accordance with the manufacturer’s guidelines. The concentration values were reported in pg/ml.

***Cell culture***

In DMEM medium (Hyclone, Beijing, China) supplemented with 10% FBS (Hyclone, Beijing, China) and 1% streptomycin/penicillin, human endometrial cell lines (HEC-1B, RL95-2, and Ishikawa) were cultured at 37 ^°^C with 5% CO_2_ (31).

***Cell transfection***

Small interference RNA (siRNA) of LOXL2 (si-LOXL2) was procured from Genepharma (Shanghai, China). LOXL2 overexpression plasmid (pCMV-HMGB1) was procured from Miaolingbio (Wuhan, China). LOXL2 overexpression was performed by plasmid pCDH-CMV-LOXL2 (OE-LOXL2) and its empty vector pCDH-CMV (LMAI Bio). Transient transfection was conducted using Lipofectamine 3000 reagent (Invitrogen, USA) and followed the manufacturer’s instructions. RT-qPCR and western blot verified the transfection efficiency at 48 hr after transfection.

***3-(4,5-dimethylthiazol-2-yl)-2,5-diphenyltetrazolium bromide (MTT) assay***

In 96-well plates, 1×10^4^ cells per well were used to seed HEC-1B, RL95-2, and Ishikawa cells. Subsequently, the cells were incubated for 4 hr at 37 ^°^C with MTT solution (1 mg/ml, M1020, Solarbio, Beijing, China). Following incubation, 100 μl/well of DMSO was used to dissolve the formazan crystals. Lastly, a microplate reader was used to measure the absorbance at 570 nm in accordance with the developer’s instructions.

***Cell migration assay***

The migratory capabilities of human endometrial cells were investigated using the Transwell test. Transwell chambers (8 μm, 1×10^5^ cells/ml) were filled with cells in 100 μl DMEM medium (without FBS) for the test. Six hundred microliters of 10% FBS DMEM medium were added to the bottom container. Following a 72-hour incubation period, a cotton swab was used to clean the cells in the upper container, and 0.1% crystal violet was used to identify the moving cells. To determine the average number of moved cells, the labeled moving cells from five fields were counted under a light microscope (magnification, x100)(32).

***Cell apoptosis***

Human endometrial cells were grown in a 24-well plate with 5×10^4^ cells per well and treated for 48 hr. Following treatment, the cells were trypsin digested and then stained for 15 min at room temperature using propidium iodide (PI) and Annexin V-FITC. The rate of cell apoptosis was determined using flow cytometry by examining the Annexin V-FITC-positive and PI-negative cells. The apoptotic cells were quantified using the Annexin V-FITC assay kit (CA1020, Solarbio, Beijing, China) in accordance with the developer’s instructions.

***Immunofluorescence***

The Immunofluorescence technique was performed using the method described previously (33). Frozen human EC cells were washed three times with phosphate-buffered saline (PBS) after they were collected. The cells were then treated with TUNEL (C1086, Beyotime), DAPI (S0063, Beyotime), and Mito Ferro Green (HY-D2295; MedChemExpress) stains and examined under an inverted microscope (IX51, Olympus, Japan).

***Real-time quantitative PCR (RT-qPCR)***

The PrimeScript RT Reagent Kit (TaKaRa Bio Inc, Dalian, China) was used to synthesize cDNA from 1 μg of total RNA extracted from HEC-1B, RL95-2, and Ishikawa cells. RNA was isolated using TRIzol (Invitrogen). A Takara SYBR Green RT-qPCR kit and an Applied Biosystems 7500 real-time PCR machine (CA, USA) were used to perform RT-qPCR. For this study, LOXL2 primer sequences were used (forward: 5′-CTG CAA GTT CAA TGC CGA GT-3′; reverse: 5′-TCT CCA CCA GCA CCT CCA CTC-3′). GAPDH was utilized as an internal reference. The NCBI Primer-Blast Tool (https://www.ncbi.nlm.nih. gov/tools/primer-blast/) was used to design the LOXL2 primer. However, the RT-qPCR settings included 7 min of denaturation at 95 ^°^C, 40 cycles of 15 sec at 95 ^°^C, and 1 minute at 60 ^°^C. The relative mRNA level was estimated using the 2^-ΔΔCt^ technique (32).

***Western blotting***

In the experiment, nuclear protein extraction was carried out using Extraction Reagents (Pierce Biotechnology, Inc., Rockford, IL, USA), and total protein isolation was accomplished using RIPA lysis buffer. The protein concentration was determined using a BCA protein assay kit (Beyotime Biotechnology, China). After being separated using 10% SDS-PAGE, the 50 μg protein samples were transferred to PVDF membranes. After blocking the membranes with 5% low-fat milk, they were incubated with primary antibodies LOXL2 (1:500, ab154904, rabbit polyclonal, Abcam), SLC7A11 (1:500, ab175186, rabbit monoclonal, Abcam), FTH1 (1:500, sc-376594, mouse monoclonal, Santa Cruz), GPX4 (1:500, sc-166570, mouse monoclonal, Santa Cruz), p-PI3K (Tyr458)(1:500, ab278545, rabbit monoclonal, Abcam), PI3K (1:500, ab302958, rabbit monoclonal, Abcam), p-Akt (Ser473)(1:500, ab81283, rabbit monoclonal, Abcam), and Akt (1:500, ab8805, rabbit polyclonal, Abcam). After that, HRP-linked secondary antibodies were applied to the membranes, and GAPDH (1:2000, ab8245, mouse monoclonal, Abcam) was utilized as the internal control. Ultimately, ECL (Thermo, Waltham, MA, USA) was used to observe the bands, and ImageJ was used to analyze them.

***Statistical analysis***

The study utilized the SPSS 26.0 software package as the statistical tool. For categorical variables, percentages were utilized, and mean±standard deviation was employed to represent continuous variables. The two groups were compared using the Student’s t-test, and multiple comparisons were made using one-way ANOVA. A statistically significant difference was indicated by *P*<0.05.

## Results

***LOXL2 expression was elevated in EC tissues, and serum***

**Figure 1:**
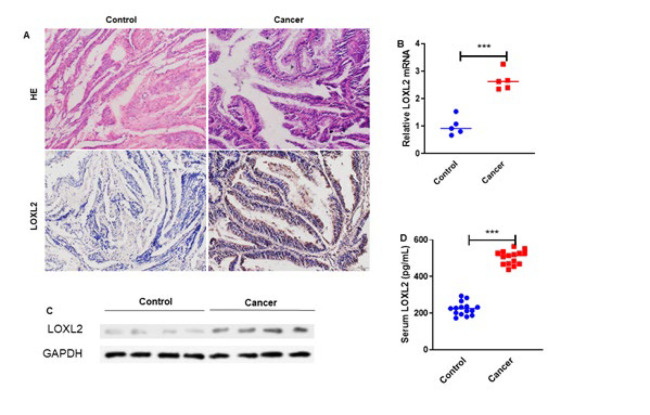
LOXL2 expression was up-regulated in endometrial cancer tissues and serum

This experiment aimed to explore the expression pattern of LOXL2 in EC tissues and serum. We performed HE staining and LOXL2 expression by immunohistochemistry in EC and normal adjacent tissues to achieve this. We found that LOXL2 expression was significantly increased in EC tissues compared to normal adjacent tissues (Figure 1A). We performed RT-qPCR analysis to determine the mRNA expression of LOXL2 in EC and normal adjacent tissues to validate the observations. We found that LOXL2 expression was up-regulated in EC tissues (*P*<0.001)(Figure 1B).

Additionally, western blot analysis was conducted to determine the protein expression of LOXL2 in EC tissues (n=5) and normal adjacent tissues (n=5), and the results indicated that LOXL2 expression levels were elevated in EC tissues (Figure 1C). Moreover, the serum concentrations of LOXL2 from 15 EC patients and 15 healthy controls were measured using ELISA. The findings illustrated a notable elevation in LOXL2 expression within the serum of patients diagnosed with EC, with statistical significance demonstrated at a level of *P*<0.001 (Figure 1D). Therefore, the above findings speculate that LOXL2 expression is up-regulated in EC tissues and serum.

***Silencing LOXL2 in EC cells inhibits cell proliferation and migration***

**Figure 2:**
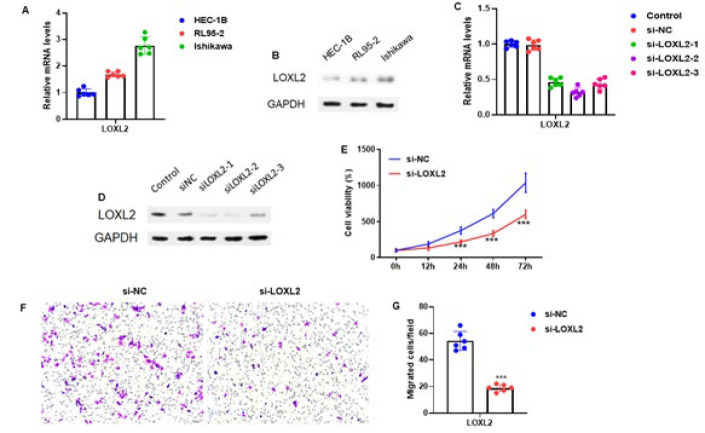
Silencing of LOXL2 in EC cells suppresses cell proliferation and migration

The mRNA expression of LOXL2 in three EC cell lines (HEC-1B, RL95-2, and Ishikawa) was analyzed using RT-qPCR. When Ishikawa cells were compared to two other EC cell lines (HEC-1B and RL95-2), the results revealed that LOXL2 expression levels were higher in Ishikawa cells (Figure 2A). In addition, the western blot analysis was performed to determine the protein expression of LOXL2 in these three EC cell lines. The same consistent expression pattern of LOXL2 was observed in Ishikawa cells (Figure 2B). Next, we chose Ishikawa cells transfected with three sequences of si-LOXL2 or control si-NC and cultured them for 48 hours. We performed the RTqPCR analysis to assess mRNA levels of LOXL2. We observed that LOXL2 expression was reduced in three sequences of si-LOXL2 (Figure 2C).

Additionally, the western blot analysis was employed to assess protein levels of LOXL2 in transfected Ishikawa cells, and the consistent expression pattern in three sequences of si-LOXL2 was found (Figure 2D). We then performed the MTT assay to determine the cell viability in Ishikawa cells transfected with si-LOXL2 and si-NC for 12, 24, 48, and 72 hr. Moreover, we did not observe any adverse cell viability issues (Figure 2E). Furthermore, the Transwell assay evaluated the migration ability of Ishikawa cells transfected with si-LOXL2 and si-NC for 24 hr. Cells were stained with 0.1% crystal violet (magnification 100×). The results demonstrated that the migration ability of Ishikawa cells transfected with si-LOXL2 was reduced (Figure 2F). After quantification of the number of migrated cells, we observed the same consistently reduced migration ability of the Ishikawa cells transfected with si-LOXL2 (*P*<0.001) (Figure 2G). Therefore, these results suggest that silencing LOXL2 in EC cells inhibits cell proliferation and migration.

***Silencing LOXL2 increases the apoptosis of EC cells***

**Figure 3:**
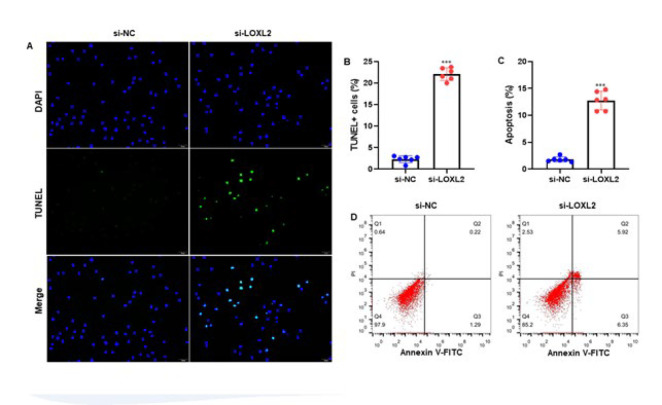
Silencing of LOXL2 enhances EC cell apoptosis. Ishikawa cells were transfected with siRNA targeting LOXL2 mRNA or control siRNA for 24 hr

Ishikawa cells were transfected with siRNA targeting LOXL2 mRNA or control siRNA for 24 hr to assess the impact of silencing LOXL2 on EC cell apoptosis. Cells were stained with TUNEL and DAPI and were observed and photographed under a fluorescence microscope. The results showed that LOXL2 silencing increased EC cell apoptosis (Figure 3A). After quantification of the percentage of TUNEL-positive cells relative to DAPI-positive cells, an elevated apoptosis rate was observed in EC cells (*P*<0.001) (Figure 3B). Additionally, Annexin V-FITC double staining was used to measure, and flow cytometry was used to analyze cell apoptosis. The apoptotic rate was computed as the product of the upper and lower right quadrant. The findings indicated that LOXL2 silencing raised EC cell apoptosis (*P*<0.001) (Figure 3C and D). Thus, the above findings indicate that silencing LOXL2 elevated the apoptosis of EC cells.

***Silencing LOXL2 promotes EC cell ferroptosis***

**Figure 4:**
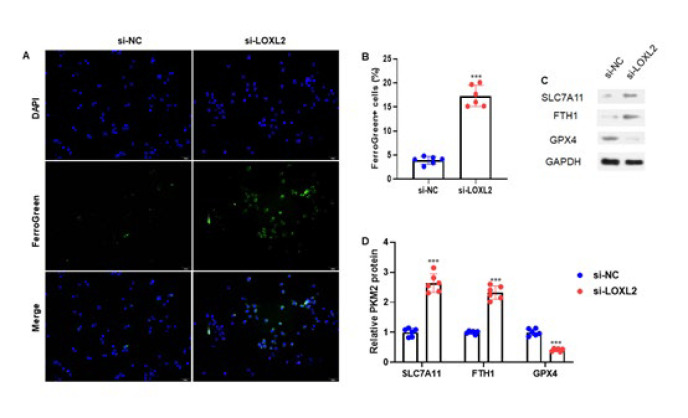
Silencing of LOXL2 enhances EC cell ferroptosis

The experiments investigated the potential effect of LOXL2 silencing on EC cell ferroptosis. For 24 hr, Ishikawa cells were transfected with either control or LOXL2 mRNA-targeting siRNA. Cells were then stained with Mito FerroGreen to evaluate mitochondrial ferrous iron. The results showed increased ferroptosis in EC cells (Figure 4A). After quantification of the percentage of FerroGreen positive cells relative to DAPI positive cells, the same elevated ferroptosis rate in the EC cells was found (*P*<0.001) (Figure 4B). Additionally, three ferroptosis-related proteins, including Solute Carrier Family 7 Member 11 (SLC7A11), Ferritin Heavy Chain 1 (FTH1), and Glutathione Peroxidase 4 (GPX4), were assessed in Ishikawa cells treated with si-LOXL2 or si-NC using western blot analysis. We observed that SLC7A11 and FTH1 expressions were increased while GPX4 was decreased compared to si-NC (Figure 4C). After quantification, we observed the consistent expression pattern of SLC7A11, FTH1, and GPX4 proteins (both *P*<0.001)(Figure 4D). Therefore, the experiment results suggest that LOXL2 silencing elevated EC cell ferroptosis.

***LOXL2 protects EC cells against ferroptosis by activating the PI3K/Akt pathway***

**Figure 5:**
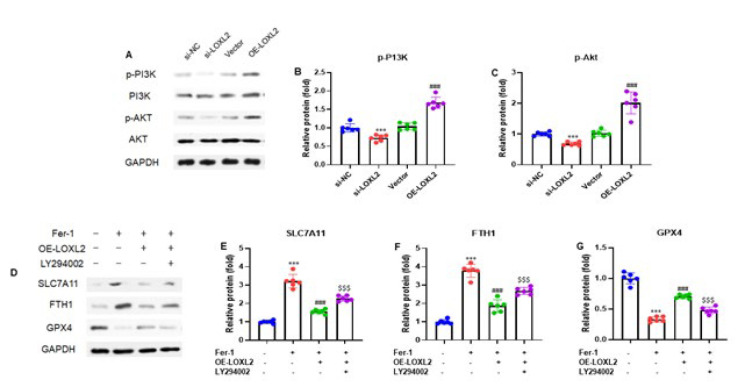
LOXL2 protects EC cells from ferroptosis by activating the PI3K/Akt pathway

We conducted a western blot analysis to explore LOXL2’s protective mechanism against ferroptosis. Representative bands of PI3K/Akt pathway proteins were observed in Ishikawa cells with LOXL2 silencing or overexpression. The results showed that LOXL2 silencing reduced the p-PI3K and p-Akt protein expression, while LOXL2 overexpression (OE-LOXL2) elevated the p-PI3K and p-Akt protein expression (Figure 5A). After quantification of the protein bands, we observed the same trends of the p-PI3K and p-Akt protein expression (*P*<0.001)(Figure 5B and C). Additionally, Ishikawa cells were transfected with vector pCDH-CMV (OE-LOXL2) and or treated with ferrostatin-1 (fer-1, 1 μM) and PI3K inhibitor LY294002 (10 μM) for 24 hr. We then performed the western blot analysis to evaluate the expression levels of the ferroptosis-related proteins such as SLC7A11, FTH1, and GPX4 in Ishikawa cells and Ishikawa cells treated with fer-1 and LY294002. The experimental data indicated that LOXL2 silencing elevated the expression levels of SLC7A11 and FTH1, while GPX4 expression was decreased. On the other hand, LOXL2 overexpression reduced the SLC7A11 and FTH1 protein expression and increased the GPX4 protein expression. However, LY294002 treatment restored the elevated SLC7A11 and FTH1 expression and decreased the expression of GPX4 in Ishikawa cells (Figure 5D). After quantification, we found the same consistent expression pattern of SLC7A11, FTH1, and GPX4 in Ishikawa cells (*P*<0.001)(Figures 5E, F, and G). Thus, the above results demonstrated that LOXL2 protects EC cells from ferroptosis by activating the PI3K/Akt pathway.

## Discussion

In our research, we studied the possible impact of LOXL2 on EC cells’ survival, migration, and ferroptosis. Our findings showed that the expression of LOXL2 was higher in EC cells. By silencing LOXL2 in EC cells, we observed a reduction in cell proliferation and migration, an increase in apoptosis, and an elevation in EC cell ferroptosis. Furthermore, we discovered that LOXL2 activates the PI3K/Akt pathway to protect EC cells from ferroptosis. These results suggest that LOXL2 might be a potential target for treating EC.

According to a previous study, LOXL2, which belongs to the LOX gene family, can cross-link extracellular matrix collagen and elastin. This extracellular matrix remodeling makes tumors more susceptible to distant metastasis and local infiltration (34). Earlier research has found a strong association between cancer progression and dysregulation of LOXL2 (35). LOXL2 expression has also been detected in the serums of patients with idiopathic pulmonary fibrosis (36) and rheumatoid arthritis-associated interstitial lung disease (37). Furthermore, LOXL2 expression was positively correlated with atrial fibrosis in patients with atrial fibrillation (38). In patients with endometriosis-associated infertility, high LOX expression was observed in the endometrial epithelium, and LOX overexpression led to increased gene expression related to fibrosis and ECM remodeling (39). However, our study found that the expression levels of LOXL2 were significantly higher in EC tissues and serum compared to normal adjacent tissues.

To better understand the function of LOXL2 in EC, we measured the expression of LOXL2 mRNA in three different cell lines: HEC-1B, RL95-2, and Ishikawa. The results showed that Ishikawa cells had higher levels of LOXL2 expression compared to the other two cell lines. We then selected Ishikawa cells, transfected them with either three sequences of si-LOXL2 or control siRNA (si-NC), and cultured them for 48 hr. The results showed that the silencing of LOXL2 significantly influenced cell proliferation and migration. Additionally, the capacity of tumor cells to undergo apoptosis was enhanced considerably by the silencing of LOXL2. These findings suggest that LOXL2 plays a crucial role in developing EC cells.

Ferroptosis, a novel form of cell death, relies on iron and plays a crucial role in amino acid and iron metabolism, oxidative stress, and tumor suppression (6-9). It is closely related to several types of cancer, including liver, stomach, pancreatic, breast, and ovarian cancers (10-15). Ferroptosis activation could be a promising approach to treating most tumors (16). However, in this study, Ishikawa cells were transfected with siRNA that targeted LOXL2 mRNA or control siRNA for 24 hr. It was discovered that ferroptosis was significantly affected in EC cells. Following this, the three ferroptosis-related proteins (SLC7A11, FTH1, and GPX4) in Ishikawa cells were treated with si-LOXL2 or si-NC. The results indicated that SLC7A11 and FTH1 expressions were increased while GPX4 was decreased compared to si-NC. Therefore, the experiment results suggest that LOXL2 silencing elevated EC cell ferroptosis. 

Wu *et al*. reported that aberrant expression of LOXL2 was revealed in liver malignancies. By activating the PI3K/AKT signaling pathway, LOXL2 can improve hepatocellular carcinoma cell infiltration and invasion and promote metastasis (40). However, this study aimed to elucidate the relationship between LOXL2, ferroptosis, and the PI3K/AKT signaling pathway. To do this, PI3K, AKT, p-PI3K, and p-AKT expression levels were investigated in cell models with LOXL2 silenced and LOXL2 overexpressed. The results show that following LOXL2 silencing, p-PI3K and p-AKT expression levels were significantly lower, and following LOXL2 overexpression, they were elevated. Furthermore, Ishikawa cells were treated with ferrostatin-1 (fer-1, 1 μM) and PI3K inhibitor LY294002 (10 μM) for 24 hr and transfected with vector pCDH-CMV (OE-LOXL2). The experimental data showed that LOXL2 silencing increased the expression levels of SLC7A11 and FTH1 while decreasing GPX4 expression. Conversely, LOXL2 overexpression decreased SLC7A11 and FTH1 protein expression and increased GPX4 protein expression. However, LY294002 treatment restored the elevated SLC7A11 and FTH1 expression and reduced the expression of GPX4 in Ishikawa cells. Therefore, these findings suggest that LOXL2 protects EC cells from ferroptosis by activating the PI3K/Akt pathway. 

(A) HE staining and LOXL2 expression by immunohistochemistry were performed in endometrial cancer tissues and adjacent normal tissue (Magnification 200×). (B) RT-qPCR was carried out to determine the mRNA expression of LOXL2 in endometrial cancer tissues and adjacent normal tissue. (C) Western blot was carried out to determine the protein expression of LOXL2 in EC tissues (n=5) and normal adjacent tissues (n=5). (D) ELISA was carried out to measure the serum concentrations of LOXL2 from EC patients (n=15) and healthy controls (n=15). Data are expressed as mean±SD in triplicates (n=6) and analyzed using the Student t-test. ***P<0.001 vs control group

EC; endometrial cancer

(A) mRNA expression of LOXL2 in three EC cell lines (HEC-1B, RL95-2, and Ishikawa) by RTqPCR. (B) Western blot was carried out to determine the protein expression of LOXL2 in these three EC cell lines. Ishikawa cells were transfected with LOXL2 siRNA (three sequences of si-LOXL2) or control siRNA (si-NC) and were cultured for 48 hr. (C) RTqPCR was performed to assess mRNA levels of LOXL2. (D) Western blot was performed to assess protein levels of LOXL2. (E) Cell viability was determined by MTT assay in Ishikawa cells transfected with si-LOXL2 and si-NC for 12, 24, 48, and 72 hr. (F) Transwell assay was used to assess the migration ability of Ishikawa cells transfected with si-LOXL2 and si-NC for 24 hr. Cells were stained with 0.1% crystal violet (magnification 100×). (G) Quantification of the number of migrated cells. Data are expressed as mean±SD in triplicates (n=6) and analyzed using the Student t-test. ***P<0.01 vs si-NC group

EC; endometrial cancer

(A) Cells were stained with TUNEL and DAPI and observed and photographed under a fluorescence microscope (magnification 200×). (B) Quantification of the percentage of TUNEL-positive cells relative to DAPI-positive cells. (C) Cell apoptosis was assessed by Annexin V-FITC double staining and analyzed by flow cytometry. (D) The representative images of flow cytometry for apoptosis. The apoptotic rate was calculated by the sum of the lower right quadrant and the upper right quadrant. Data are expressed as mean ± SD in triplicates (n = 6) and analyzed using the Student t-test. ***P<0.001 vs si-NC group

EC; endometrial cancer

(A) Ishikawa cells were stained with Mito FerroGreen to evaluate mitochondrial ferrous iron. Representative images of FerroGreen positive cells are shown (magnification 200×). (B) Quantification of the percentage of FerroGreen positive cells relative to DAPI positive cells. (C) Western blot was performed to assess three ferroptosis-related proteins, SLC7A11, FTH1, and GPX4, in Ishikawa cells treated with si-LOXL2 or si-NC. (D) Quantification of SLC7A11, FTH1, and GPX4 protein. Calculations of all these proteins were normalized to GAPDH. Data are expressed as mean±SD (n=6) and analyzed using the Student t-test. ***P<0.001 vs si-NC group

EC; endometrial cancer

(A) Representative PI3K/Akt pathway protein bands by western blot in Ishikawa cells with LOXL2 silencing or overexpression. (B, C) Quantification of p-PI3K (normalized to total PI3K protein) and p-Akt (normalized to total Akt protein) protein. ***P<0.001 vs si-NC group; ^###^*P<*0.001 vs Vector group. Ishikawa cells were transfected with vector pCDH-CMV (OE-LOXL2) and/or treated with ferrostatin-1 (fer-1, 1 μM) and PI3K inhibitor LY294002 (10 μM) for 24 hr. (D) Representative bands of ferroptosis-related proteins by western blot in Ishikawa cells. (E, F, G) Quantification of SLC7A11, FTH1, and GPX4 in Ishikawa cells treated with fer-1 and LY294002. ***P<0.001 vs control group (fer-1, OE-LOXL2 and LY294002, all negative); ^###^*P<*0.001 vs fer-1 group; ^$$$^*P<*0.001 vs fer-1+OE-LOXL2 group

EC; endometrial cancer

## Conclusion

The research findings indicate that LOXL2 phosphorylates PI3K and AKT, activating the PI3K/AKT signaling pathway, which significantly increases the progression of EC cells, including survival, migration, and ferroptosis. Therefore, LOXL2 could be a potential therapeutic target for treating EC. In the future, we will verify our results in *in vivo* settings.

## Data Availability

Due to confidential issues, the datasets generated and/or analyzed during the current work are not publicly available but are available from the corresponding author upon reasonable request.

## Declaration

We have not used any AI tools or technologies to prepare this manuscript.

## Limitations

It is worth noting that this study has several constraints. Firstly, the number of EC cell samples we examined is relatively low, and the pathological types we focused on are only EC. Expanding the number of tissue samples and pathological types of EC cells would be better. Additionally, we only investigated the effects of LOXL2 in EC cells *in vitro*. Thus, additional research is required to validate LOXL2’s effects on EC cells *in vivo*.
